# Synthesizing 30-years of adult medicaid dental policy research: A scoping review to identify gaps and opportunities

**DOI:** 10.1016/j.heliyon.2023.e13703

**Published:** 2023-02-13

**Authors:** Jason Semprini, Riley Samuelson

**Affiliations:** a University of Iowa, College of Public Health, Department of Health Management and Policy, USA; b University of Iowa, College of Dentistry, USA; c University of Iowa Hardin Library of Health Sciences, USA

**Keywords:** Medicaid, Disparities, Healthcare reform, Policy evaluation, Review

## Abstract

**Objective:**

Despite the importance of Medicaid for the oral health of low-income adults, the extent to which Medicaid dental policy variation influences outcomes is unknown. This study aims to review the evidence evaluating adult Medicaid dental policies to synthesize conclusions and motivate future research.

**Data sources:**

A comprehensive search of academic literature published in English between 1991 and 2020 was conducted to identify studies which evaluated an adult Medicaid dental policy for its effect on outcomes. Studies strictly involving children, policies not related to adult Medicaid dental coverage, and non-evaluation studies were excluded. The data analysis identified the policies, outcomes, methods, populations, and conclusions of the included studies.

**Results:**

Among the 2731 unique articles extracted, 53 met the inclusion criteria. 36 studies evaluated the effect of expanding Medicaid dental coverage, which was found to consistently increase dental service visits (21 studies) and reduce unmet dental needs (4 studies). Provider density, reimbursement rates, and level of benefits appear to influence the effect of expanding Medicaid dental coverage. The evidence for changing Medicaid benefits and reimbursement rates were mixed for its impact on provider participation and emergency dental services. Few studies examined how adult Medicaid dental policies impact health outcomes.

**Conclusions:**

Most of the recent research has focused on evaluating the effect of expanding or reducing Medicaid dental coverage on dental service utilization. Future research investigating the impact of adult Medicaid dental policies on clinical, health, and wellness outcomes remains warranted.

**Clinical significance:**

Low-income adults are responsive to Medicaid dental policy changes and utilize more care with more generous coverage. Less is known about how these policies influence health.

## 1 Introduction

For decades, Medicaid has served as a critical source of oral health access for low-income adults in the United States, albeit with comprehensive coverage variability [[1], [2], [3]]. Medicaid is a safety net health insurance program that is jointly funded by federal and state governments, but implemented by the states. This unique design gives states flexibility to operate their Medicaid program by setting eligibility guidelines and covering different health benefits. While most states provide some level of adult Medicaid dental coverage, the range of benefits varies widely by state and has changed over time [[4], [5], [6]]. Despite the importance of Medicaid for the oral health of low-income adults, the extent to which Medicaid dental policy variation influences outcomes is unknown. A complete understanding of the known effects of Medicaid dental policy to-date can improve evidence-based policy diffusion, as well as expand the oral health research agenda.

With the goal of influencing future Medicaid dental research, this scoping review [7] aims to synthesize the research evaluating adult Medicaid dental policies to identify the policies, populations, methodologies, and outcomes analyzed over the past thirty years and to explore any evidence gaps to motivate future research opportunities.

## 2 Review questions

What is the nature of adult Medicaid dental policy research over the past thirty years?

What conclusions can be drawn from the evidence?

What adult Medicaid dental policies were studied?

What adult populations were included in the analyses?

What datasets and methodologies were utilized?

What outcomes were assessed?

What evidence gaps remain for future research evaluating adult Medicaid dental policies?

What policies should be evaluated for their effect on adult oral health outcomes?

What specific populations have yet to be appropriately investigated?

What datasets and methodologies could improve the field of research?

What additional outcomes have yet to be explored?

What research findings remain mixed, unclear, or unanswered?

## 3 Inclusion criteria

### 3.1 Participants

This review considered studies that included low-income adults who may or may not have been eligible for or enrolled in Medicaid, as well as dental professionals participating or not participating in state Medicaid programs.

### 3.2 Concept

This review considered studies that evaluated the effect of adult Medicaid dental policies on outcomes.

#### 3.2.1 Context

This review considered studies that were published in English between 1991 and 2020. Peer-reviewed publications, as well as white papers and theses were considered for inclusion.

### 3.3 Types of sources

This scoping review considered quantitative, qualitative, and mixed methods study designs. Because this review focuses on policy evaluation, included studies were required to meet a minimal degree of validity and scientific rigor. Quantitative and qualitative studies were considered only if the design a) included a comparator group (i.e. pre/post, treatment/control) or b) utilized a quasi-experimental or natural experiment design. Research designs with only a single group, at a single time-point without any attempt to control for internal validity issues were excluded from the review. Additionally, this review only considered retrospective evaluations, and excluded analyses solely projecting future outcomes.

## 4 Methods

This scoping review was conducted in accordance with the JBI methodology for scoping reviews [8], following the PRISMA-extension for scoping reviews [9].

### 4.1 Search strategy

The search strategies were developed in collaboration with a health sciences librarian trained in systematic searching. The search strategies aimed to locate scholarly published primary studies. All identified keywords, synonyms, and controlled vocabulary (when available) were adapted for each information source. The full search strategies are provided in Table 1. The searches were conducted in September 2020; English language filters were utilized in each database. Studies published in English between January 1, 1991 and September 15, 2020 were included. We investigated this thirty year period given the variability of adult Medicaid dental programs during this time [[4], [5], [6]]. The databases that were searched included: PubMed, Embase, Scopus, CINHAL, PQ Dissertations and Theses, Ageline, and ABI/INFORM Global [[10], [11], [12], [13], [14], [15], [16]]. Sources of unpublished studies and gray literature were outside the scope of this review.

**Table 1:** Search strategy conducted on September 16, 2020.

Database	Query	Records retrieved
PubMed	("Medicaid"[Mesh] OR “Medicaid”[tw] OR “Title 19”[tw] OR "Centers for Medicare and Medicaid Services, U.S."[Mesh] OR "Centers for Medicare and Medicaid Services, U.S."[tw]) AND ("Oral Health"[Mesh] OR “oral Health”[tw] OR "Dental Facilities"[Mesh] OR "Dental Clinics"[Mesh] OR "Dental Clinics"[tw] OR "Dental Clinic"[tw] OR “dental office”[tw] OR “dental offices”[tw] OR "Dental Health Services"[Mesh] OR "Dental Health Services"[tw] OR "Dental Health Service"[tw] OR "Dental Care"[Mesh] OR "Dental Care"[tw] OR "Dentistry"[Mesh] OR “dentistry”[tw] OR “dental”[tw] OR "Periodontics"[Mesh] OR "Periodontics"[tw] OR "Periodontic"[tw] OR "Periodontal"[tw] OR "Endodontics"[Mesh] OR "Endodontic"[tw] OR “endodontics”[tw] OR “endodontology”[tw] OR "Surgery, Oral"[Mesh] OR "oral surgery"[tw] OR “exodontics”[tw] OR “exodontic”[tw] OR “Maxillofacial Surgery”[tw] OR "Orthodontics"[Mesh] OR "Orthodontics"[tw] OR "Orthodontic"[tw] OR "Dentists"[Mesh] OR "Dentists"[tw] OR “dentist”[tw] OR “Prosthodontists”[tw] OR “Prosthodontist”[tw] OR “Periodontists”[tw] OR “Periodontist”[tw] OR "Oral and Maxillofacial Surgeons"[Mesh] OR "Oral and Maxillofacial Surgeons"[tw] OR "Oral and Maxillofacial Surgeon"[tw] OR "Oral Surgeons"[tw] OR "Oral Surgeon"[tw] OR “Maxillofacial Surgeons"[tw] OR “Maxillofacial Surgeon"[tw] OR “exodontist”[tw] OR “exodontists”[tw] OR "Endodontists"[Mesh] OR "Endodontists"[tw] OR "Endodontist"[tw] OR "Orthodontists"[Mesh] OR "Orthodontists"[tw] OR "Orthodontist"[tw] OR "Dental Service, Hospital"[Mesh] OR "Dental Service, Hospital"[tw] OR “Hospital Dental Service”[tw] OR “Hospital Dental Services”[tw] OR "Mouth Rehabilitation"[Mesh] OR "Mouth Rehabilitation"[tw] OR "Mouth Rehabilitations"[tw])	1782
Embase (09/16/20)	('medicaid'/exp OR ‘Medicaid’:ab,ti,kw OR ‘Title 19’:ab,ti,kw OR ‘Centers for Medicare and Medicaid Services, U.S.’:ab,ti,kw) AND (‘oral Health’:ab,ti,kw OR 'dental facility'/exp OR 'dental clinic'/exp OR ‘Dental Clinics’:ab,ti,kw OR ‘Dental Clinic’:ab,ti,kw OR ‘dental office’:ab,ti,kw OR ‘dental offices’:ab,ti,kw OR 'dental procedure'/exp OR ‘Dental Health Services’:ab,ti,kw OR ‘Dental Health Service’:ab,ti,kw OR ‘Dental Care’:ab,ti,kw OR 'dentistry'/exp OR ‘dentistry’:ab,ti,kw OR ‘dental’:ab,ti,kw OR 'periodontics'/exp OR ‘Periodontics’:ab,ti,kw OR ‘Periodontic’:ab,ti,kw OR ‘Periodontal’:ab,ti,kw OR 'endodontics'/exp OR ‘Endodontic’:ab,ti,kw OR ‘endodontics’:ab,ti,kw OR ‘endodontology’:ab,ti,kw OR 'oral surgery'/exp OR ‘oral surgery’:ab,ti,kw OR ‘exodontics’:ab,ti,kw OR ‘exodontic’:ab,ti,kw OR ‘Maxillofacial Surgery’:ab,ti,kw OR 'orthodontics'/exp OR ‘Orthodontics’:ab,ti,kw OR ‘Orthodontic’:ab,ti,kw OR 'dentist'/exp OR ‘Dentists’:ab,ti,kw OR ‘dentist’:ab,ti,kw OR ‘Prosthodontists’:ab,ti,kw OR ‘Prosthodontist’:ab,ti,kw OR ‘Periodontists’:ab,ti,kw OR ‘Periodontist’:ab,ti,kw OR 'dental surgeon'/exp OR ‘Oral and Maxillofacial Surgeons’:ab,ti,kw OR ‘Oral and Maxillofacial Surgeon’:ab,ti,kw OR ‘Oral Surgeons’:ab,ti,kw OR ‘Oral Surgeon’:ab,ti,kw OR ‘Maxillofacial Surgeons’:ab,ti,kw OR ‘Maxillofacial Surgeon’:ab,ti,kw OR ‘exodontist’:ab,ti,kw OR ‘exodontists’:ab,ti,kw OR 'endodontist'/exp OR ‘Endodontists’:ab,ti,kw OR ‘Endodontist’:ab,ti,kw OR 'orthodontist'/exp OR ‘Orthodontists’:ab,ti,kw OR ‘Orthodontist’:ab,ti,kw OR ‘Dental Service, Hospital’:ab,ti,kw OR ‘Hospital Dental Service’:ab,ti,kw OR ‘Hospital Dental Services’:ab,ti,kw OR 'full mouth rehabilitation'/exp OR ‘Mouth Rehabilitation’:ab,ti,kw OR ‘Mouth Rehabilitations’:ab,ti,kw)	1770
CINAHL (09/16/20)	((MH "Medicaid+") OR (“Medicaid”) OR (“Title 19”) OR (MH "United States Centers for Medicare and Medicaid Services") OR ("Centers for Medicare and Medicaid Services, U.S.")) AND ((MH "Oral Health") OR (“oral Health”) OR (MH "Dental Facilities+") OR (MH "Dental Clinics") OR ("Dental Clinics") OR ("Dental Clinic") OR (“dental office”) OR (“dental offices”) OR (MH "Dental Health Services+") OR ("Dental Health Services") OR ("Dental Health Service") OR (MH "Dental Care+") OR ("Dental Care") OR (MH "Dentistry+") OR (“dentistry”) OR (“dental”) OR (MH "Periodontics+") OR "Periodontics") OR ("Periodontic") OR ("Periodontal") OR (MH "Endodontics+") OR ("Endodontic") OR (“endodontics”) OR (“endodontology”) OR (MH "Surgery, Oral+") OR ("oral surgery") OR (“exodontics”) OR (“exodontic”) OR (“Maxillofacial Surgery”) OR (MH "Orthodontics+") OR ("Orthodontics") OR ("Orthodontic") OR (MH "Dentists+") OR ("Dentists") OR (“dentist”) OR (“Prosthodontists”) OR (“Prosthodontist”) OR (“Periodontists”) OR (“Periodontist”) OR ("Oral and Maxillofacial Surgeons") OR ("Oral and Maxillofacial Surgeon") OR ("Oral Surgeons") OR ("Oral Surgeon") OR (“Maxillofacial Surgeons") OR (“Maxillofacial Surgeon") OR (“exodontist”) OR (“exodontists”) OR (MH "Endodontists") OR ("Endodontists") OR ("Endodontist") OR ("Orthodontists") OR ("Orthodontist") OR ("Dental Service, Hospital") OR (“Hospital Dental Service”) OR (“Hospital Dental Services”) OR ("Mouth Rehabilitation") OR ("Mouth Rehabilitations"))	1205
Scopus (09/16/20)	(TITLE-ABS-KEY(“Medicaid” OR “Title 19” OR “Centers for Medicare and Medicaid Services, U.S.”) OR INDEXTERMS(“medicaid”)) AND (TITLE-ABS-KEY(“oral Health” OR “Dental Clinics” OR “Dental Clinic” OR “dental office” OR “dental offices” OR “Dental Health Services” OR “Dental Health Service” OR “Dental Care” OR “dentistry” OR “dental” OR “Periodontics” OR “Periodontic” OR “Periodontal” OR “Endodontic” OR “endodontics” OR “endodontology” OR “oral surgery” OR “exodontics” OR “exodontic” OR “Maxillofacial Surgery” OR “Orthodontics” OR “Orthodontic” OR “Dentists” OR “dentist” OR “Prosthodontists” OR “Prosthodontist” OR “Periodontists” OR “Periodontist” OR “Oral and Maxillofacial Surgeons” OR “Oral and Maxillofacial Surgeon” OR “Oral Surgeons” OR “Oral Surgeon” OR “Maxillofacial Surgeons” OR “Maxillofacial Surgeon” OR “exodontist” OR “exodontists” OR “Endodontists” OR “Endodontist” OR “Orthodontists” OR “Orthodontist” OR “Dental Service, Hospital” OR “Hospital Dental Service” OR “Hospital Dental Services” OR “Mouth Rehabilitation” OR “Mouth Rehabilitations”) OR INDEXTERMS(“dental facility” OR “dental clinic” OR “dental procedure” OR “dentistry” OR “periodontics” OR “endodontics” OR “oral surgery” OR “orthodontics” OR “dentist” OR “dental surgeon” OR “endodontist” OR “orthodontist” OR “full mouth rehabilitation”)	1910
Ageline (09/16/20)	(“Medicaid” OR “Title 19” OR “Centers for Medicare and Medicaid Services, U.S.”) AND (“oral Health” OR “Dental Clinics” OR “Dental Clinic” OR “dental office” OR “dental offices” OR “Dental Health Services” OR “Dental Health Service” OR “Dental Care” OR “dentistry” OR “dental” OR “Periodontics” OR “Periodontic” OR “Periodontal” OR “Endodontic” OR “endodontics” OR “endodontology” OR “oral surgery” OR “exodontics” OR “exodontic” OR “Maxillofacial Surgery” OR “Orthodontics” OR “Orthodontic” OR “Dentists” OR “dentist” OR “Prosthodontists” OR “Prosthodontist” OR “Periodontists” OR “Periodontist” OR “Oral and Maxillofacial Surgeons” OR “Oral and Maxillofacial Surgeon” OR “Oral Surgeons” OR “Oral Surgeon” OR “Maxillofacial Surgeons” OR “Maxillofacial Surgeon” OR “exodontist” OR “exodontists” OR “Endodontists” OR “Endodontist” OR “Orthodontists” OR “Orthodontist” OR “Dental Service, Hospital” OR “Hospital Dental Service” OR “Hospital Dental Services” OR “Mouth Rehabilitation” OR “Mouth Rehabilitations”)	78
ProQuest Dissertations & Theses Global (09/16/20)	(“Medicaid” OR “Title 19” OR “Centers for Medicare and Medicaid Services, U.S.”) AND (“oral Health” OR “Dental Clinics” OR “Dental Clinic” OR “dental office” OR “dental offices” OR “Dental Health Services” OR “Dental Health Service” OR “Dental Care” OR “dentistry” OR “dental” OR “Periodontics” OR “Periodontic” OR “Periodontal” OR “Endodontic” OR “endodontics” OR “endodontology” OR “oral surgery” OR “exodontics” OR “exodontic” OR “Maxillofacial Surgery” OR “Orthodontics” OR “Orthodontic” OR “Dentists” OR “dentist” OR “Prosthodontists” OR “Prosthodontist” OR “Periodontists” OR “Periodontist” OR “Oral and Maxillofacial Surgeons” OR “Oral and Maxillofacial Surgeon” OR “Oral Surgeons” OR “Oral Surgeon” OR “Maxillofacial Surgeons” OR “Maxillofacial Surgeon” OR “exodontist” OR “exodontists” OR “Endodontists” OR “Endodontist” OR “Orthodontists” OR “Orthodontist” OR “Dental Service, Hospital” OR “Hospital Dental Service” OR “Hospital Dental Services” OR “Mouth Rehabilitation” OR “Mouth Rehabilitations”)	149
ABI/INFORM Global (09/16/20)	(“Medicaid” OR “Title 19” OR “Centers for Medicare and Medicaid Services, U.S.”) AND (“oral Health” OR “Dental Clinics” OR “Dental Clinic” OR “dental office” OR “dental offices” OR “Dental Health Services” OR “Dental Health Service” OR “Dental Care” OR “dentistry” OR “dental” OR “Periodontics” OR “Periodontic” OR “Periodontal” OR “Endodontic” OR “endodontics” OR “endodontology” OR “oral surgery” OR “exodontics” OR “exodontic” OR “Maxillofacial Surgery” OR “Orthodontics” OR “Orthodontic” OR “Dentists” OR “dentist” OR “Prosthodontists” OR “Prosthodontist” OR “Periodontists” OR “Periodontist” OR “Oral and Maxillofacial Surgeons” OR “Oral and Maxillofacial Surgeon” OR “Oral Surgeons” OR “Oral Surgeon” OR “Maxillofacial Surgeons” OR “Maxillofacial Surgeon” OR “exodontist” OR “exodontists” OR “Endodontists” OR “Endodontist” OR “Orthodontists” OR “Orthodontist” OR “Dental Service, Hospital” OR “Hospital Dental Service” OR “Hospital Dental Services” OR “Mouth Rehabilitation” OR “Mouth Rehabilitations”)	143
Total Records		7037

### 4.2 Study/source of evidence selection

Following the search, all identified records were collated and uploaded into EndNote Desktop [17]. After removing duplicates, titles and abstracts were screened for assessment against the inclusion criteria for the review. Potentially relevant papers were retrieved, and those full-text articles that did not meet the inclusion criteria were excluded. The reasons for their exclusion are provided in Appendix 1. Appendix 2 contains the reference list for all studies included in the analysis.

### 4.3 Data extraction and analysis

Data were extracted from papers included in the scoping review in accordance with the research objectives. First, the policy of interest was identified for each primary study. Then, data were extracted to identify the specific populations studied. This segment also indicated whether the study included subgroups in the analyses. The extraction process then identified the research methodology and datasets used to evaluate the specific policy. Next, each outcome of the study was identified. Finally, each study's main conclusion and implication was summarized.

## 5 Results

### 5.1 Study inclusion

**Fig. 1:**
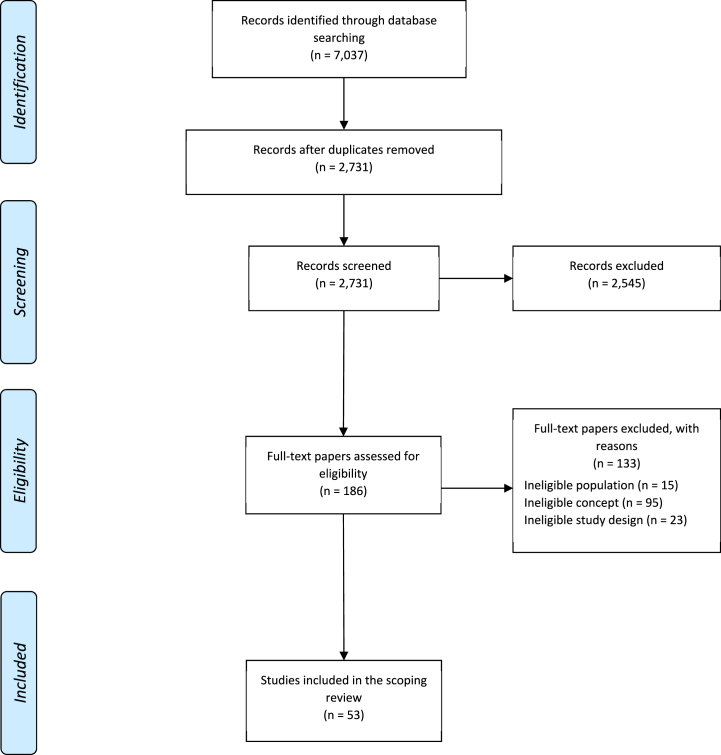
Search results and study selection and inclusion process.

The search identified 7037 distinct records, from which 2731 were unique, non-duplicate primary scholarly studies. 2545 studies were excluded by screening the title and abstract. Among the 186 full texts reviewed, 15 were excluded for studying an ineligible population (i.e., children), 95 were excluded for an ineligible concept (i.e., not evaluating Medicaid dental policies), and 23 were excluded for lacking a minimum level of study design, resulting in 53 studies meeting the inclusion criteria (Fig. 1).

### 5.2 Summary and characteristics of included studies

Each of the 53 studies met the inclusion criteria for evaluating an Adult Medicaid dental policy between 1991 and 2020 [[18], [19], [20], [21], [22], [23], [24], [25], [26], [27], [28], [29], [30], [31], [32], [33], [34], [35], [36], [37], [38], [39], [40], [41], [42], [43], [44], [45], [46], [47], [48], [49], [50], [51], [52], [53], [54], [55], [56], [57], [58], [59], [60], [61], [62], [63], [64], [65], [66], [67], [68], [69], [70]]. Supplemental Table 1 provides a tabulated overview for all 53 studies. In addition to providing each paper's title, author list, and year of publication, Supplemental Table 1 reports the 1) policy evaluated, 2) populations studied, 3) methodology and dataset used to conduct the research, 4) outcomes studied. Supplemental Table 1 table concludes with a brief summary of each study's primary conclusions and research findings. Table 2, Table 3, Table 4, Table 5, Table 6 report the counts of policies, populations, methodologies, datasets, and outcomes of the studies included in this review.

**Table 2:** List of medicaid policies.

Policy	n
ACA Medicaid Expansion	21
Full Medicaid Dental Coverage	6
Change in Medicaid Dental Benefits	6
Eliminating Medicaid Reimbursement for Emergency Dental Services	5
Early Medicaid Expansion	2
Health behavior incentives for Medicaid dental program	2
Medicaid Dental Reimbursement Rates	2
Annual Medicaid Dental Limits	1
Eliminating non-emergency Medicaid dental reimbursement	1
Integrated Medicaid Managed Care	1
Levels of Adult Medicaid Dental Coverage	1
Medicaid Dental Accountable Care Organizations	1
Medicaid Managed Care	1
Medicaid priority lists	1
Medicaid State Plan Amendments	1
Reducing Medicaid dental reimbursement rate	1
Medicaid Budget Cuts and Administrative Requirements	1

**Table 3:** List of populations.

Population	n
Adult Medicaid Beneficiaries	28
*State Specific Medicaid Beneficiaries*	21
*Continuously Enrolled Medicaid Beneficiaries*	4
*Treated at Academic Medical Center*	*5*
*w/Subgroups*	*8*
Low-Income Adults	11
*State Specific Low-Income Adults*	*4*
*Treated at Academic Medical Center*	*1*
*w/Subgroups*	*5*
Providers	4
Categorically Eligible or Population Specific	3
Population Estimates	1

**Table 4:** List of analytical methodologies.

Methodology	n
Descriptive or Retrospective Analyses	19
Difference-in-Differences	13
Regression Models	13
Panel Methods (Two-way Fixed Effects)	7
Propensity Score Methods	4
Instrumental Variable Methods	4
Interrupted Time Series	4
Generalized Equations	2

**Table 5:** List of datasets.

Dataset	n
Administrative Data	32
*State-Specific Administrative Data*	*18*
*Population Administrative Data*	*9*
*Hospital-Specific Administrative Data*	*5*
Secondary Survey Data	16
*Behavioral Risk Factor Surveillance System*	*9*
*Gallup-Healthways Wellbeing Survey*	*2*
*Medical Expenditure Panel Survey*	*2*
*National Health and Nutritional Examination Survey*	*1*
*Health Center Patient Survey*	*1*
*American Dental Association*	*1*
*National Health Interview Survey*	*1*
Primary Survey Data	5

**Table 6:** List of outcomes.

Outcomes	n
Dental Service Visits	21
Emergency/Hospital Dental Visits	15
Dental Needs/Difficulties Obtaining Care	9
Dental Expenditures (by patient)	5
Provider Participation in Medicaid	5
Dental Costs to Provider	5
Specialty Dental Visits	3
Dental Prescriptions	2
Reimbursement Rates	1
Socioeconomic Dental Inequality	1
Untreated Caries	1
Missing or Broken Teeth	1
Wait Times	1
Tooth Saving Services	1
Tooth Extractions	1

Medicaid Expansion was the most frequently studied policy (21 studies). The most common population studied were Adult Medicaid beneficiaries (28 studies). Thirty-two studies analyzed administrative data. Among the studies attempting to infer causality, Differences-in-Differences designs were the most common approach (15 studies). Dental service visits were the most frequently used outcome variable among the studies in this review (21 studies).

The following section summarizes the conclusions from the articles included in this review and is organized by the policy studied. The section on policies changing Medicaid dental coverage includes subsections for outcomes studied.

## 6 Conclusions from articles included in the scoping review

### 6.1 Changing medicaid dental coverage

#### 6.1.1 Dental service visits & access

The studies evaluating expanding Medicaid dental coverage, either by expanding Medicaid before the ACA [19,45], expanding Medicaid through the ACA [18,[22], [23], [24],[30], [31], [32],^36^,[38], [39], [40],^43^,^50^,^51^,^53^,^60^,^62^,^64^,^65^,^68^,^70^], or increasing the level of adult Medicaid dental benefits [20,28,29,41,49], found unequivocal evidence that dental coverage expansion increased access to dental care. Thirteen studies found increased dental visits [22,24,28,29,32,40,49,51,62,64,68,70] and four studies found reduced unmet dental care needs [19,31,45,60].

Dental Medicaid coverage was also found to increase prescriptions for dental conditions [45],utilization of specialized services [54], out-of-pocket expenditures [65], and dental care wait times [20]. Of particular interest, many of these effects were influenced by external factors or policies. Regarding the increase in wait times for dental care, the effect of expanding Medicaid dental coverage appeared to only increase wait times in states with more restrictive dental assistant scope-of-practice laws [20]. Additionally, dental services use increased after Medicaid dental expansion, but only for states with higher reimbursement rates [28,32] and in states with higher levels of benefit coverage [40,41,68]. Further, Medicaid dental expansion was found to have no effect on dental service visits in areas with lower dental provider density [68] and fewer federally qualified health centers [37].

##### 6.1.1.1 Emergency-department services

The evidence for Medicaid dental coverage's impact on non-traumatic emergency or hospital-based dental services, however, appears to be less consistent. 3 studies suggest expanding coverage reduces non-traumatic emergency dental services [30,38,39], while 4 studies suggest the opposite, that expansion leads to increased emergency dental services [23,28,29,35].

##### 6.1.1.2 Reimbursement rates and dental provider behavior

Regarding the impact on providers, one study found that Medicaid dental expansion led to lower Medicaid reimbursement rates [43]. This could explain the finding from another study which showed that provider participation decreased after Medicaid expansion [70]. However, another study found that Medicaid expansion, in fact, increased the number of providers participating in Medicaid dental programs [20].

#### 6.1.2 Clinical health and disparities

From a clinical perspective, 1 study found that Medicaid dental coverage reduced untreated caries and broken or missing teeth [29]. However, regarding socioeconomic disparities in oral health, there was no evidence that expanding Medicaid dental coverage reduced the racial, gender, or age gaps in dental services use [36].

### 6.2 Eliminating reimbursement for emergency dental services

Multiple studies in this review evaluated a Maryland policy which eliminated Medicaid reimbursement for dentists who provide emergency dental services [[25], [26], [27],^48^]. The investigators of these studies exploited the fact that reimbursement rates to hospitals and emergency departments for dental emergency services were not affected by the policy change. Evidence consistently suggested that patients merely substituted away from dentists to emergency departments and hospitals for non-traumatic dental care. One of these studies also found that out-of-pocket expenditures for the patient, costs to the provider, and expenses to the state increased because of eliminating reimbursement to dentists for emergency services [48]. Unmet dental care needs also appeared to increase [48].

### 6.3 Changing medicaid dental reimbursement rates

Among the studies evaluating the effect of changing Medicaid dental reimbursement rates, one study found that reducing reimbursement rates led to lower non-traumatic emergency dental services [54]. However, the associated costs of providing non-traumatic emergency dental services increased [54]. However, a second study found lowering reimbursement rates increased non-traumatic emergency dental services [51]. Another study evaluated the effect of increasing reimbursement rates on provider participation, and the subsequent effect on preventative dental service visits [59]. This study found that the increase in provider participation from rising reimbursement rates are considerably greater than the associated effect on dental services utilization. Specifically, a 10% increase in reimbursement rates corresponds with a 10% increase in provider participation, but only leads to a minimal (∼1%) increase in dental service visits.

### 6.4 Other demand-side and service policy changes

Reducing, but not eliminating Medicaid dental benefits was consistently found to increase non-traumatic dental services in emergency departments or hospitals [35,52,55]. Benefit reductions were also found to increase unmet dental needs [67]. The study estimating the effect of annual benefit limits found no effect on dental services use on the general Medicaid population, but did however, observe a major reduction in services for dual-eligible beneficiaries and beneficiaries with a diagnosed disability [47].

The studies evaluating changes to the Medicaid dental program found that preventative dental service utilization was higher for Medicaid managed care [21], integrated managed care [69], and accountable care organizations [66]. Policies which created an incentive system for accessing dental benefits, however, were found to increase perceived barriers to care, while having no effect on provider participation [56,57].

## 7 Discussion

Most of the studies in this review evaluated the effect of expanding Medicaid dental coverage. This is especially common in the past ten years, where most of the studies involved the ACA's Medicaid Expansion. Generally, these studies evaluated the effect of the ACA Medicaid Expansion on dental service utilization on Medicaid beneficiaries or low-income adults. As a result, the field of Medicaid dental research can and must expand. Based on the policies, populations, data/methods, and outcomes of the studies included in this review, the following section discusses gaps and opportunities for future Medicaid dental research, with the goal of improving health outcomes.

### 7.1 Recommendations for future research

#### 7.1.1 Policies

With strong evidence suggesting that expanding Medicaid coverage increases utilization, the field can begin to identify policies which may be intensifying or suppressing this effect. Such research has already been discussed in this review, but other heterogeneity may be present. Meanwhile, few studies investigated the impact of different models of service delivery despite the continued proliferation of Medicaid managed care plans for state dental programs [71,72]. The cost-sharing schemes and provider network contracts have yet to be fully assessed for their impact on dental health utilization for low-income adults. Additionally, research studying how policies influence various dimensions of dental service access remain warranted. Based on the Andersen model of healthcare access, health care must be available, appropriate, acceptable, affordable [73]. These dimensions of access have been given less attention from the studies included in this review. Only one study considered the effect of dental assistant scope of practice laws as a moderating effect following dental expansion. No study, at least in the past thirty years, has investigated any variation due to policies directly addressing provider shortages in Medicaid programs. State incentive schemes repaying dental student loan debt should be further explored for variation between and within states, and subsequently evaluated for their short- and long-term efficacy. Medicaid teledentistry is another avenue that remains to be evaluated for its effect on dental care availability. And, while the level of Medicaid benefits was heavily studied, less attention was given to the variation in services covered by states providing Medicaid dental benefits. Such variation can be extremely critical when evaluating the appropriateness of dental care and the unmet dental needs of low-income adults. A simple cross-section shows the stark heterogeneity in services available between states [4]. Such research could not only improve the field's understanding of the effect of appropriate access and different levels of treatment intensity but inform future cost-effectiveness and cost-benefit analyses for appropriately allocating adult Medicaid dollars. Policies addressing public and social determinants were also largely ignored. As states continue to identify and address social determinants, dental health researchers must contribute by identifying evidence-based policies to integrate oral health within policies improving overall health and wellness [74].

### 7.2 Populations

Low-income adults are themselves a population of interest, given the exceptional dental needs and adverse outcomes facing the group. But, even within this group, disparities persist. Older adults, non-Hispanic Black adults, adults with a history of smoking, and adults with a chronic disease have all been found to be at increased risk of poor dental health [[75], [76], [77], [78]]. Rurality creates an additional layer of inequity. Not only are rural adults more likely to be poorer, sicker, and older (and more likely to smoke), but rural adults living in lower resource areas have fewer dental providers and live further from dental specialists [79,80]. While many of the included studies analyzed subgroups of their target population of interest, these subgroups were typically constrained to age, gender, and racial differences. Rurality and parental status were also studied, but less frequently. This is simply not enough to inform policies attempting to mitigate oral health disparities.

### 7.3 Data and methodology

Researchers are confined by the availability of data. Data constraints pose additional problems for disparities research, as by definition, there are fewer minorities (racial, ethnic, rural) in the population, and therefore fewer minorities in publicly available and restricted data [[81], [82], [83]]. Yet, at least for rural populations, methodological frameworks exist for improving small-n survey research [84]. Similar frameworks should be further developed and utilized in oral health research for minorities shouldering the burden of poor oral health.

Most of the data in this review came from restricted sources, which can be expensive and burdensome to obtain. Conversely, among the articles included in our review, primary data were rare, likely due to the cost of implementing interviews, focus groups, and surveys. These costs hinder the ability of lower resource investigators from contributing meaningful advances to the field. Policies which reduce these costs may not increase the dissemination of Medicaid dental policy research but improve equity and diversity within oral health science.

Regarding analytical approaches, while most of the studies did not attempt to infer causation, over the past ten years the studies included in this review were more likely to attempt an approach identifying a policy's effect. The most used approach were Differences-in-Differences designs. This simple design can provide great insights into observational studies and provide a potentially valid estimate of a policy's effect or association on an outcome. But, just as Medicaid dental policies have evolved over thirty years, so too have econometric methods and our understanding about Difference-in-Differences designs [85]. Hopefully the oral health research community will continue to incorporate advanced methods to both support conclusions from traditional approaches, as well as facilitate new evidence to improve our understanding of how Medicaid policies impact health outcomes. Such analytically rigorous methods can also help researchers tease out unobserved confounding factors to improve our understanding of the mechanisms driving oral health inequities, as well as provide stronger identification strategies to find the true effect of our policy of interest.

In addition to identification and statistical approaches, the field of oral health research would also stand to benefit from the renewed rigor of legal epidemiological methods [86]. Such systematic policy scan approaches can fundamentally expand the extent of Medicaid policies investigated in a manner which encourages reproducibility and continual policy evaluation.

### 7.4 Outcomes

Dental service visits, either preventative or non-traumatic emergency care, were the most heavily studied outcomes for research included in this review. Given the dearth of evidence for the effect of Medicaid dental policies on clinical or health status outcomes, researchers should be motivated to fil this critical gap. Policymakers will benefit from knowing whether or not certain approaches to Medicaid implementation are in fact improving oral health and overall wellbeing.

Three other opportunities are now discussed to motivate a broader lens for the potential impact of oral health research. The United States is facing three ongoing health crises, each with a high level of mortality and morbidity fueled by underlying health inequities. The first is the nation's opioid crisis [87]. Research has begun to establish a relationship between dentistry prescribing practices with opioid overdoses across the country [[88], [89], [90]]. Yet, no studies have attempted to investigate whether any Medicaid policies protected or exacerbated these affects. Such research would be timely and immediately inform the efforts to end the opioid crisis.

The second crisis relates to the ongoing disparities, both rural and racial, for cancer mortality and survival. While not as common as the more prevalent cancers, oral cancer continues to increase death and disability across the population. Despite the disease burden, oral cancer can be prevented through behavior change and mortality can be reduced considerably by early detection [91,92]. Dentists are often on the front lines of both efforts, but disparities persist [93]. Medicaid expansion, teledentistry, scope of practice laws, and tobacco cessation programs in dental clinics should be heavily investigated for their effect on oral cancer. Research has also supported the claim that survival improves for patients completing a comprehensive dental exam prior to radiation treatment for Head and Neck cancer [94]. The connection between Medicaid dental practices and survival in this deadly cancer cannot be understated, and has the potential be cost-saving and efficient.

Finally, the impact of the covid-19 disaster on our health and economic wellbeing will no doubt linger for years to come. Dentistry and oral health will not be immune to such effects. Questions related to the delay of oral health services during the early months of lockdown could improve our understanding on the value of Medicaid dental services, as will the stark rise in telehealth provide an opportunity to evaluate different models and contexts of Medicaid teledentistry delivery. As the long-term impacts on healthcare and public health budgets materialize, the policy decisions related to Medicaid financing and service delivery could reshape the landscape of dental care delivery for low-income adults. Rapid policy identification, evaluation, and dissemination has the potential to prevent adverse outcomes for patients, providers, and the public.

## 8 Conclusion

This review synthesizes clear, unambiguous evidence that expanding adult Medicaid dental benefits leads to increased dental visits. This effect, however, appears to be influenced by the density of dental providers, state reimbursement rates, and level of state Medicaid dental benefits. Along with confirming the long-term effect of Medicaid dental coverage on preventative and emergency visits, there appears to be great opportunity for researchers aiming to investigate the impact of more nuanced Medicaid dental policies on clinical, health, and wellness outcomes using advanced research designs. Given the increased implementation of state Medicaid waivers and amendments, as well as managed care and cost-sharing policies, such evidence could inform continual development of evidenced-based oral health policies. While the dearth of publicly available data on dental policies and oral health outcomes may be hindering the progress of adult Medicaid dental research, legal epidemiology and advanced causal inference methodologies highlight a path to advance the adult Medicaid dental research agenda.

## Author contribution statement

All authors listed have significantly contributed to the development and the writing of this article.

## Funding statement

Mr. Jason Semprini was supported by 10.13039/100000072National Institute of Dental and Craniofacial Research [T90 DE023520-08].

## Data availability statement

No data was used for the research described in the article.

## Declaration of competing interest

The authors declare no conflict of interest.
