# Supplementary material for: Synthesizing 30-years of adult medicaid dental policy research: A scoping review to identify gaps and opportunities
Source: Heliyon. 2023 Feb 13;9(2):e13703. doi: 10.1016/j.heliyon.2023.e13703 (PMC9975108; doi:10.1016/j.heliyon.2023.e13703)
Supplement: Multimedia component 1 [file mmc1.pdf]

Supplemental Table 1: Characteristics of included studies

Ref. No.	Study	Policy	Population	Methodology	Dataset	Outcome	Conclusion
18	AlSagob, E. I. (2017). "Non-Traumatic Dental Visits to Hospital-Based Emergency Departments in Rhode Island"	ACA Medicaid Expansion	RI Adults visiting Emergency Department; subgroup analyses by gender, race, age, and metro-status.	Interrupted Time-Series	RI Hospital Discharge Data	Non-Traumatic Dental Visits in Emergency Departments	Medicaid Expansion caused an immediate increase in Non-Traumatic Dental Visits in Emergency Departments
19	Baicker, K., H. L. Allen, B. J. Wright, S. L. Taubman and A. N. Finkelstein (2018). "The Effect of Medicaid on Dental Care of Poor Adults: Evidence from the Oregon Health Insurance Experiment."	Early Medicaid Expansion	Low-income adults in OR who participated in a lottery to receive Medicaid coverage;	Randomization Instrumental Variable Analysis	OR Emergency Department Records, Primary Survey Data	Dental care needs; Dental care utilization; Emergency Department Visits for dental care; Dental care medications; Out-of-Pocket costs for dental care	Medicaid expansion reduced unmet dental health needs by increasing emergency department visits and medication use. No effect on out-of-pocket spending.
20	Buchmueller, T., S. Miller and M. Vujicic (2016). "How do providers respond to changes in public health insurance coverage? Evidence from adult medicaid dental benefits."	Full Medicaid Dental Coverage, Dental Assistant Scope of Practice	Random Sample of Dentists	Two-Way Fixed Effect Linear Regression; Instrumental Variable Two-Stage Least Squares Regression	Survey of Dental Practices (ADA)	Medicaid participation; Medicaid receipts; Total Medicaid visits (by type of visit); Labor and Price indicators; Wait Times	Expanding Medicaid dental coverage increases dentists' participation in Medicaid and the number of patients seen with Medicaid, increases total visits and labor supply, and increases wait times especially for states with limited scope of provider laws (dental assistants/hygienists). with restrictive scope of practice laws governing dental hygienists
21	Burns, M. E. (2009). "Medicaid managed care and cost containment in the adult disabled population."	Medicaid Managed Care	Working, Adult Disabled Population	Two-Part Panel regression with lagged county-level controls, and state and time fixed effects.	Medical Expenditure Panel Survey	Medicaid expenditures	The probability of any dental expenditures and the level of expenditures are higher for adults in counties with voluntary Medicaid managed care plans compared to fee-for-service. Living in a county with mandatory or voluntary managed care leads to a positive and significant average partial effect on dental expenditures.
22	Cawley, J., A. Soni and K. Simon (2018). "Third Year of Survey Data Shows Continuing Benefits of Medicaid Expansions for Low-Income Childless Adults in the U.S."	ACA Medicaid Expansion	Low-income Childless Adults (HHI < 100% FPL)	Difference-in-Differences Linear Probability Model	Behavioral Risk Factor Surveillance System	Dental visit in the past 12 months	Medicaid expansion increased the probability of visiting the dentist in the past year.
23	Chalmers, N., J. Grover and R. Compton (2016). "After medicaid expansion in Kentucky, use of hospital emergency departments for dental conditions increased."	ACA Medicaid Expansion	KY Adult Medicaid beneficiaries, with sex subgroups by age and race subgroups by rurality.	Pooled and Panel w/ 2-Way Fixed Effects Multivariate Logistic Regression Models with Rare Event Correction	State Emergency Department Databases of the Healthcare Cost and Utilization Project	Dental and oral health condition emergency department discharges	Medicaid expansion increased dental related emergency discharges, with higher increases for male and non-white adults.
24	Choi, M. K. (2011). "The impact of Medicaid insurance coverage on dental service use."	ACA Medicaid Expansion	Low-income Adults (HHI < $10,000), comparing childless and parents	Difference-in-Differences Linear Probability Model	Behavioral Risk Factor Surveillance System	Dental visit in past year	Medicaid expansion increased the probability of visiting the dentist in the past year, for both parents and childless adults.
25	Cohen, L. A., R. J. Manski and F. J. Hooper (1996). "Does the elimination of Medicaid reimbursement affect the frequency of emergency department dental visits?"	Eliminating Medicaid Reimbursement for Emergency Dental Services	MD Adult Medicaid beneficiaries who used ED services at an academic medical center, with sex and race subgroups	Descriptive and Chi-2 Analyses	Hospital Discharge Data	Emergency visits for dental conditions	Eliminating Medicaid reimbursement for dentists providing emergency services led to an increase in emergency dental visits at hospitals.
26	Cohen, L. A., R. J. Manski, L. S. Magder and C. D. Mullins (2002). "Dental visits to hospital emergency departments by adults receiving Medicaid: assessing their use."	Eliminating Medicaid Reimbursement for Emergency Dental Services	MD Adult Medicaid beneficiaries who used ED services with sex, race, and age subgroups	Multivariable Poisson Regression (Natural Experiment)	Maryland Medicaid Information System	Emergency department dental visits	Eliminating Medicaid reimbursement for dentists providing emergency services led to an increase in dental visits in hospital emergency departments.
27	Cohen, L. A., R. J. Manski, L. S. Magder and C. D. Mullins (2003). "A Medicaid Population's Use of Physicians' Offices for Dental Problems."	Eliminating Medicaid Reimbursement for Emergency Dental Services	MD Adult Medicaid beneficiaries who used physician services with sex, race, and age subgroups	Rate ratio analyses (Natural Experiment)	Maryland Medicaid Information System	Medicaid Claims from Physicians' Offices for Dental-Related Emergencies	Eliminating Medicaid reimbursement for dentists providing emergency services led to a decrease in emergency dental visits at physician offices.

Supplemental Table 1: Characteristics of included studies

Ref. No.	Study	Policy	Population	Methodology	Dataset	Outcome	Conclusion
28	Decker, S. L. and B. J. Lipton (2015). "Do Medicaid benefit expansions have teeth? The effect of Medicaid adult dental coverage on the use of dental services and oral health."	Full Medicaid Dental Coverage, Medicaid Dental Reimbursement Rates	Low-income Adult Medicaid beneficiaries,	Triple Differences Linear Probability Model (using income as the third difference)	National Health Interview Survey, National Health and Nutrition Examination Survey	Dental visit in the past six months, Dental visit in the past twelve months, Untreated dental caries, broken or missing teeth.	Medicaid dental coverage increases the probability of a recent dental visit, with the effect increasing with higher reimbursement rates. Medicaid dental coverage may also reduce the likelihood of untreated dental caries and broken or missing teeth.
29	Doan, L., T. Tiwari, D. Brunson and C. M. Carey (2017). "Medicaid Adult Dental Benefit Impact on Dental Utilization: A University Clinic Setting."	Full Medicaid Dental Coverage	CO Adult Medicaid beneficiaries using services at an academic medical center.	Descriptive and Chi-2 Analyses.	University of Colorado School of Medicine Dental Records	Dental visits by type of service, number of patients receiving dental services.	Expanding Medicaid dental coverage substantially increased the number of patients receiving dental related services. Expansion increased the number of tooth saving services, while decreasing the number of tooth extractions.
30	Elani, H. W., I. Kawachi and B. D. Sommers (2020). "Changes in emergency department dental visits after Medicaid expansion."	ACA Medicaid Expansion	Non-elderly adults residing in low-income counties who used hospital services.	Differences-in-Differences Linear Regression Model	State Emergency Department Database	Emergency visits for dental conditions, Medicaid coverage for dental services	Medicaid Expansion reduced emergency department dental visits and increased the Medicaid coverage for adults seeking dental care.
31	Farietta, T. P., B. Lu and R. Tumin (2018). "Ohio's Medicaid Expansion and Unmet Health Needs Among Low-Income Women of Reproductive Age."	ACA Medicaid Expansion	OH Women of Reproductive Age	Survey-weighted logistic regression model	Ohio Medicaid Assessment Survey	Unmet dental care needs, dental visits	Medicaid expansion reduced the odds of an unmet dental care need, but had no effect on the odds of visiting a dentist in the past year.
32	Ferdousi, W. (2017). “Inequality in Access to Dental and Vision Care: Examining the Role of Income and Insurance”	ACA Medicaid Expansion, Medicaid Dental Reimbursement Rates	Low-income Childless Adults	Differences-in-Differences Linear Probability Model with Two-Way fixed effects, Triple Differences Linear Probability Model (childless adults as third difference).	Behavioral Risk Factor Surveillance System	Dental Visit in the past twelve months	Medicaid Expansion increased the probability of visiting a dentist in the past year, with minimally increasing effects with higher reimbursement rates.
33	Fingar, K. R., M. W. Smith, S. Davies, K. M. McDonald, C. Stocks and M. C. Raven (2015). "Medicaid dental coverage alone may not lower rates of dental emergency department visits."	Full Medicaid Dental Coverage, dental provider density	County-level estimates, by rural/urban status	Descriptive, t-tests, and multivariate regression models for rate data	State Emergency Department Databases	Emergency department visits for nontraumatic dental conditions	Medicaid dental coverage may not reduce dental ED visits in areas of low provider density and rural regions.
34	Grover, S. S. (2013). “Minnesota medicaid budget cutbacks 2010 and impact on dental service utilization in nursing home residents”	Change in Medicaid Dental Benefits	Adult Medicaid beneficiaries, older than age 55, who received dental services in an academic medical nursing facility.	Retrospective observational analyses	University of Minnesota Community-based Nursing Facility data	Dental services in a nursing facility	Reducing Medicaid dental benefits may decrease dental service utilization for both Medicaid beneficiaries and older adults without public insurance receiving care in nursing facilities.
35	Katz, S. (2011). "The individual and program impacts of eliminating Medicaid dental benefits in the Oregon Health Plan."	Change in Medicaid Dental Benefits	OR Medicaid beneficiaries, continuously enrolled on standard plan.	Differences-in-Differences with propensity score matching.	Oregon Health Plan Survey data, Oregon Health Plan administrative data	Unmet dental needs, preventative dental visits in the past year, ambulatory dental visits	Eliminating dental benefits increased the likelihood of reporting an unmet dental need, reduced the likelihood of receiving preventative services in the past year, and increased the use of emergency ambulatory care for dental conditions.
36	Kino, S. and I. Kawachi (2018). "The impact of ACA Medicaid expansion on socioeconomic inequality in health care services utilization."	ACA Medicaid Expansion	Adults between age 18-64.	Differences-in-Differences Weighted Linear Regression Model	Behavioral Risk Factor Surveillance System	Socioeconomic inequalities in dental visits	While the ACA Medicaid Expansion reduced inequalities in many healthcare services, there was no effect on inequalities in dental visits.
37	Kirksey, V. (2019). “Assessing the Impact of South Carolina's Medicaid Adult Dental Policy on Dental Emergency Department Visits”	Full Medicaid Dental Coverage	SC Adult Medicaid beneficiaries living in the community	Multivariable logistic regression	SC Medicaid claims, SC Medicaid enrollment data, SC all-payer emergency department data	Dental visits by service type and location	Adult Medicaid dental coverage reduced non-traumatic emergency dental services, with greater effects occurring in areas with a Federally Qualified Health Center.

Supplemental Table 1: Characteristics of included studies

Ref. No.	Study	Policy	Population	Methodology	Dataset	Outcome	Conclusion
38	Laniado, N., V. M. Badner and E. J. Silver (2017). "Expanded Medicaid dental coverage under the Affordable Care Act: an analysis of Minnesota emergency department visits."	ACA Medicaid Expansion	MN Adult Medicaid beneficiaries who used emergency services, with age subgroups	Descriptive and Chi-2 analyses	State Emergency Department Databases for Minnesota	Emergency dental visits for dental conditions	Expanding dental coverage through the ACA Medicaid Expansion reduced non-traumatic emergency dental visits, with the greatest impact occurring in younger adults.
39	Laniado, N., A. R. Brow, E. Tranby and V. M. Badner (2020). "Trends in non-traumatic dental emergency department use in New York and New Jersey: a look at Medicaid expansion from both sides of the Hudson River."	ACA Medicaid Expansion	NJ and NY adults who used emergency services	Descriptive and Chi-2 analyses	State Emergency Department Database of the Healthcare Cost and Utilization Project	Non-traumatic dental service discharges from emergency departments	ACA Medicaid Expansion reduced uninsured non-traumatic emergency dental services, and increased (by a larger magnitude) non-traumatic emergency dental services for Medicaid beneficiaries.
40	Lyu, W., D. M. Shane and G. L. Wehby (2020). "Effects of the Recent Medicaid Expansions on Dental Preventive Services and Treatments."	ACA Medicaid Expansion, level of state Medicaid dental coverage	Low-income adults (age 19-64)	Differences-in-Differences Event History, Linear Probability Model, with two-way fixed effects	Medical Expenditure Panel Survey	Dental services by type	Medicaid Expansion increased preventative and major dental services in states with limited or extensive dental coverage. There is less evidence for increased services in states only covering emergency dental services
41	Marthey, D. J. (2018). "Effects of Medicaid State Plan Dental Benefits on Dental Visits among Non-elderly Adults"	Levels of Adult Medicaid Dental Coverage	Adult Medicaid beneficiaries	Logistic Regression Model	Behavioral Risk Factor Surveillance System	Dental visit in the past twelve months	Higher levels of Medicaid dental coverage increase the odds of visiting the dentist in the past twelve months.
41	Marton, J., G. M. Kenney, J. E. Pelletier, J. Talbert and A. Klein (2012). "The effects of medicaid policy changes on adults' service use patterns in Kentucky and Idaho."	Medicaid State Plan Amendments (authorized by 2005 Deficit Reduction Act)	ID and KY Adult Medicaid beneficiaries, continuously enrolled on new state Medicaid managed care plan (not receiving care at rural clinic or federally qualified health center)	Logistic Regression Model	ID and KY Medicaid enrollment and claims data	Dental services by type	While these State Plan Amendment changes did not affect many health care services or outcomes, adopting Managed Care increased preventative and treatment services for dental conditions.
43	Minick, G., T. Tilliss, W. C. Shellhart, S. M. Newman, C. M. Carey, A. Horne, S. Whitt and L. J. Oesterle (2017). "Comparison of Orthodontic Medicaid Funding in the United States 2006 to 2015."	ACA Medicaid Expansion; Medicaid budget cuts	State Medicaid officials	Descriptive, pre/post analyses	Primary Survey Data	Orthodontic Medicaid Reimbursement Rates, Orthodontic Medicaid administrative requirements	Orthodontic reimbursement rates appeared to decline following ACA Expansion and 2007 Recession related budget cuts. Administrative requirements increased following the ACA Expansion and budget cuts of 2007.
44	Mitchell, J. B. and F. Bentley (2000). "Impact of Oregon's priority list on medicaid beneficiaries."	Medicaid priority lists	OR Adult Medicaid beneficiaries	Descriptive and Chi-2 analyses	Primary Survey Data	Denied dental services by reasons for denial	Priority lists increased the number of denials for dental splints, leading to an increase in services paid for out of pocket.
45	Mitchell, J. B., S. G. Haber, G. Khatutsky and S. Donoghue (2002). "Impact of the Oregon Health Plan on access and satisfaction of adults with low income."	Early Medicaid Expansion	OR low-income adults	Descriptive and Chi-2 analyses	Primary Survey Data	Unmet dental needs, dental prescription use	Among low-income adults, Medicaid dental coverage was associated with lower reports of unmet dental needs and higher use of dental prescriptions.
46	Mohamed, A., B. Alhanti, M. McCullough, K. Goodin, K. Roling and L. Glickman (2018). "Temporal association of implementation of the Arizona Health Care Cost Containment System (AHCCCS) with changes in dental-related emergency department visits in Maricopa County from 2006 to 2012."	Eliminating Medicaid Reimbursement for Emergency Dental Services	Adult Medicaid beneficiaries receiving services at a hospital in Maricopa, AZ.	General Likelihood Model (rate ratio)	Maricopa County Hospital Discharge Dataset	Hospital visits for dental-related conditions	Eliminating Medicaid reimbursement for emergency-department dental services had no impact on services, but rather changed the payer of such services (lower Medicaid, higher private pay or unreimbursed).
47	Morrison, G. C., K. S. Hendrix, G. Arling, E. Hancock, A. M. Hus, M. B. Rosenman and N. L. Swigonski (2012). "Effect of an annual benefit limit on adult dental expenditure and utilization: A cross-sectional analysis."	Annual Medicaid Dental Limits	IN Adult Medicaid Beneficiaries using dental services, with subgroup by type of Medicaid enrollment	Pre/Post Descriptive Analyses	IN Medicaid claims data	Number of dental services and Medicaid expenditures	Introducing an annual Medicaid dental limit did not impact dental services or expenditures for the Medicaid population. However, the annual limit reduced services substantially for dual-eligible and disabled Medicaid beneficiaries.

Supplemental Table 1: Characteristics of included studies

Ref. No.	Study	Policy	Population	Methodology	Dataset	Outcome	Conclusion
48	Mullins, C. D., L. A. Cohen, L. S. Magder and R. J. Manski (2004). "Medicaid coverage and utilization of adult dental services."	Eliminating Medicaid Reimbursement for Emergency Dental Services	MD Adult Medicaid beneficiaries receiving services, with age, race, and gender subgroups	Descriptive and t-test analyses	MD Medicaid Management Information System	Emergency expenditures for dental conditions	Eliminating Medicaid reimbursement to dentists for emergency services led to cost-savings for the state, but may have increased unmet dental needs and out-of-pocket costs for Medicaid beneficiaries.
49	Nasseh, K. and M. Vujicic (2013). "Health reform in massachusetts increased adult dental care use, particularly among the poor."	Full Medicaid Dental Coverage	MA Low-income adults (< 100% FPL)	Differences-in-Differences linear probability model	Behavioral Risk Factor Surveillance System	Dental visit in the past twelve months	Medicaid dental coverage increased the probability of a dental visit in the past year, with the greatest effects occurring for lower income groups.
50	Nasseh, K. and M. Vujicic (2017). "Early Impact of the Affordable Care Act's Medicaid Expansion on Dental Care Use."	ACA Medicaid Expansion	Low-income adults (< 138% FPL)	Differences-in-Differences Linear Probability Model	Gallup-Healthways Wellbeing Index Survey	Dental visits	Early evidence from ACA Medicaid Expansion doesn't indicate any change in dental visits for low-income adults.
51	Nasseh, K. and M. Vujicic (2017). "The impact of the affordable care act's Medicaid expansion on dental care use through 2016."	ACA Medicaid Expansion	Low-income adults (< 138% FPL)	Differences-in-Differences Linear Probability Model	Gallup-Healthways Wellbeing Index Survey	Dental visits	After three years of ACA Medicaid Expansion, expanded dental coverage increased dental visits for low-income adults.
52	Neely, M., J. A. Jones, S. Rich, L. S. Gutierrez and P. Mehra (2014). "Effects of cuts in Medicaid on dental-related visits and costs at a safety-net hospital."	Change in Medicaid Dental Benefits	Adults receiving care at safety-net hospital emergency department in Boston, MA, with race, age, and gender subgroups.	Retrospective Analyses	Boston Medical Center Emergency Department data	Emergency-department visits for dental conditions, cost-per visit-per year for dental emergency department visits	Reducing Medicaid dental benefits led to increased emergency department visits for dental conditions, with larger effects for older and minority adults.
53	Peck, J., C. M. Sedgley, E. Schwarz and K. J. Replogle (2019). "The impact of the Affordable Care Act on provision of endodontic services within a dental school setting in Oregon."	ACA Medicaid Expansion	OR Low-income adults (< 138 % FPL) receiving care at an academic medical center	Retrospective Analyses	Electronic Health Records at Graduate Endodontic Clinic at Oregon Health & Science University	Number of visits for endodontic treatments	ACA Expansion and Medicaid dental coverage increased endodontic services.
54	Rampa, S., F. A. Wilson, H. Wang, N. K. Wehbi, L. Smith and V. Allareddy (2018). "Hospital-Based Emergency Department Visits With Dental Conditions: Impact of the Medicaid Reimbursement Fee for Dental Services in New York State, 2009-2013."	Reducing Medicaid dental reimbursement rate	NY adults receiving dental services in an emergency department	Retrospective analyses	New York State Emergency Department Database, Health Resources and Services Administration's Area Health Resource File	Emergency department visits for dental conditions, charges for dental services in emergency department	Reducing Medicaid dental reimbursement was associated with a decline in emergency department visits and an increase in mean emergency department charges for dental conditions.
55	Ranade, A., G. Young, R. Garcia, J. Griffith, A. Singhal and J. McGuire (2020). "Changes in Dental Benefits and Use of Emergency Departments for Nontraumatic Dental Conditions in Massachusetts."	Change in Medicaid Dental Benefits	MA Adult Medicaid beneficiaries who received dental services in an emergency department.	Interrupted Time-Series Regression Model	MA All-Payer Claims Database	Non-traumatic dental services in emergency department	Eliminating full Medicaid dental benefits increased non-traumatic dental services in emergency departments. Reinstating partial Medicaid dental coverage reduced non-traumatic dental services in emergency departments.
56	Reynolds, J. C., S. C. McKernan, J. M. C. Sukalski and P. C. Damiano (2018). "Evaluation of enrollee satisfaction with Iowa's Dental Wellness Plan for the Medicaid expansion population."	Health behavior incentives for Medicaid dental program	IA Random sample of Medicaid beneficiaries newly eligible due to ACA Medicaid Expansion.	Random sample survey analysis, descriptive and Chi-2 analyses, multivariable regression analyses	Primary survey data	Plan satisfaction, ability to earn dental benefits	Many beneficiaries were not satisfied with the incentive-based Medicaid dental plan and described difficulties or lack of awareness for obtaining oral health incentives.
57	Reynolds, J. C., S. C. McKernan, P. C. Damiano and R. A. Kuthy (2019). "A tale of two public dental benefit programs: Iowa dentist participation in traditional Medicaid versus a Medicaid expansion program."	Health behavior incentives for Medicaid dental program	IA Private-practice dentists responding to a survey	Descriptive and Chi-2 analyses, binary logistic regression model	Primary survey data	Provider participation in traditional Medicaid dental program, Provider participation in incentive-based Medicaid dental program	There were no significant differences in provider participation between traditional and an incentive-based Medicaid dental programs. Dentists participating in the traditional (pre-ACA expansion enrollees) were more likely to place patient restrictions on Medicaid acceptance.

Supplemental Table 1: Characteristics of included studies

Ref. No.	Study	Policy	Population	Methodology	Dataset	Outcome	Conclusion
58	Salomon, D., R. E. Heidel, A. Kolokythas, M. Miloro and T. Schlieve (2017). "Does Restriction of Public Health Care Dental Benefits Affect the Volume, Severity, or Cost of Dental-Related Hospital Visits?"	Eliminating non-emergency Medicaid dental reimbursement	IL adults receiving care for odontogenic pain or Infection at academic medical center.	Retrospective analyses	University of Illinois Hospital Electronic Health Records	Emergency department visits for dental conditions, dental surgeries, hospital admission days due to dental conditions, complications, and cost-per patient	Eliminating non-emergency Medicaid dental reimbursement increased emergency department visits, surgeries, complications, length-of-stay, and cost-per-patient.
59	Scheffler, R. M., S. E. Foreman, P. J. Feldstein and T. W. Hu (1996). "A multi-equation model of payments and public access to services: The case of dentistry."	Medicaid dental reimbursement rates	CA dental providers and patients	Multiple Equation Weighted Least Squares regression model	Cal Dept. HHS	Provider participation in Medicaid, patient utilization for dental services	As Medicaid reimbursement rates increase, provider participation increases at a faster rate than the increase in patient service utilization. Specifically, a 10% increase in payment significantly leads to greater program participation but less than a 1% increase in utilization. yields less than 1% increase in access.
60	Seo, V., T. P. Baggett, A. N. Thorndike, P. Hull, J. Hsu, J. P. Newhouse and V. Fung (2019). "Access to care among Medicaid and uninsured patients in community health centers after the Affordable Care Act."	ACA Medicaid Expansion	Adults with continuous Medicaid coverage using services at community health centers.	Weighted Logistic Regression Model	Health Center Patient Survey	Difficulty obtaining dental care.	Medicaid expansion was associated with lower odds of reporting delaying or not receiving needed dental care.
61	Shortridge, E. F. and J. R. Moore (2009). "Use of emergency departments for conditions related to poor oral healthcare: implications for rural and low-resource urban areas for three states."	Medicaid dental reimbursement rates	UT, VT, WI Medicaid beneficiaries using emergency department, by rural and urban status	Logistic Regression Models	State Emergency Department Databases	Emergency department visits for dental conditions	Lower Medicaid reimbursement rates are associated with higher emergency department dental visits for rural Medicaid beneficiaries.
62	Simon, K., A. Soni and J. Cawley (2017). "The Impact of Health Insurance on Preventive Care and Health Behaviors: Evidence from the First Two Years of the ACA Medicaid Expansions."	ACA Medicaid Expansion	Low-income adults (< 100% FPL), with sex and parental status subgroups	Differences-in-Differences Linear Probability Model	Behavioral Risk Factor Surveillance System	Dental visit in the past twelve months	In the full sample, Medicaid Expansion was not found to impact dental visits. However, for low-income childless adults, Medicaid Expansion increased the probability of a dental visit in the past twelve months.
63	Singhal, A., D. J. Caplan, M. P. Jones, E. T. Momany, R. A. Kuthy, C. T. Buresh, R. Isman and P. C. Damiano (2015). "Eliminating medicaid adult dental coverage in California led to increased dental emergency visits and associated costs."	Change in Medicaid Dental Benefits	Adult Medicaid beneficiaries using emergency department services, with sex, race, and age subgroups.	Interrupted Time-Series linear regression model	State Emergency Department Database	Emergency department visits for dental conditions.	Eliminating Medicaid dental coverage increases emergency department visits for dental conditions. While there was no heterogeneity in this effect by race, the increase in emergency department visits were only experienced by younger adults and adults living in an urban area.
64	Singhal, A., P. Damiano and L. Sabik (2017). "Medicaid adult dental benefits increase use of dental care, but impact of expansion on dental services use was mixed."	ACA Medicaid Expansion, level of state Medicaid dental coverage	Low-income adults (< $15,000 Household Income), by parental status subgroups	Linear probability model	Behavioral Risk Factor Surveillance System	Dental visits in the past twelve months	Medicaid dental expansion led to increased dental visits for childless adults, but a decline in visits for parents.
65	Springer, R., M. Marino, J. P. O'Malley, S. Lindner, N. Huguet and J. E. Devoe (2018). "Oregon Medicaid Expenditures after the 2014 Affordable Care Act Medicaid Expansion: Over-time Differences among New, Returning, and Continuously Insured Enrollees."	ACA Medicaid Expansion	OR Adult Medicaid beneficiaries.	Inverse-propensity score weighed, restricted cubic-spline regression models	Medicaid administrative claims data	Dental expenditures	Medicaid Expansion initially increased dental expenditures, but leveled off over time.
66	Vickery, K. D., N. D. Shippee, J. Menk, R. Owen, D. M. Vock, P. Bodurtha, D. Soderlund, R. A. Hayward, M. M. Davis, J. Connett and M. Linzer (2020). "Integrated, Accountable Care For Medicaid Expansion Enrollees: A Comparative Evaluation of Hennepin Health."	Medicaid Dental Accountable Care Organizations	MN Adult Medicaid beneficiaries receiving care at Hennepin ACO or managed care organization in Minneapolis metro area. Sample included only low-income childless adults (< 75% FPL).	Two-part generalized marginal structural model with inverse-probability weighting	MN Department of Health and Human Services data	Dental visits.	Exposure to an Accountable Care Organization was associated with an increase in dental visits.

Supplemental Table 1: Characteristics of included studies

Ref. No.	Study	Policy	Population	Methodology	Dataset	Outcome	Conclusion
67	Wallace, N. T., M. J. Carlson, D. M. Mosen, J. J. Snyder and B. J. Wright (2011). "The individual and program impacts of eliminating Medicaid dental benefits in the Oregon Health Plan."	Change in Medicaid Dental Benefits	OR continuously enrolled Adult Medicaid beneficiaries.	Chi-2 analyses and multivariable logistic regression models, Differences-in-Differences linear regression model, and generalized exponential cost regression model	Oregon Health Plan Survey and Administrative data	Probability of a dental visit, Medicaid enrollment, dental expenditures, unmet dental needs.	Eliminating Medicaid dental coverage increased emergency department services and expenditures for dental conditions, unmet dental needs, and total healthcare ambulatory visits. Eliminating dental coverage also lowered the odds of a recent preventative dental visit.
68	Wehby, G. L., W. Lyu and D. M. Shane (2019). "The Impact of the ACA Medicaid Expansions on Dental Visits by Dental Coverage Generosity and Dentist Supply."	ACA Medicaid Expansion, level of state Medicaid dental coverage	Low-income adults (< 138% FPL)	Two-way fixed effects Differences-in-Differences linear probability model, with heterogeneous effects.	Behavioral Risk Factor Surveillance System	Dental visit in the past twelve months.	Expanding dental coverage increased recent dental visits, but only for states with extensive coverage and high dental supply.
69	Xiang, X., R. Owen, F. L. F. G. Langi, K. Yamaki, D. Mitchell, T. Heller, A. Karmarkar, D. French and N. Jordan (2019). "Impacts of an Integrated Medicaid Managed Care Program for Adults with Behavioral Health Conditions: The Experience of Illinois."	Integrated Medicaid Managed Care	IL Adult Medicaid beneficiaries with a behavioral health diagnosis.	Differences-in-Differences linear regression model with propensity score weights.	IL Medicaid claims, encounter, and payment data	Dental service visits	Compared to beneficiaries in enrolled in traditional Medicaid, the Integrated Medicaid Managed Care program increased dental visits for Medicaid beneficiaries with a behavioral health diagnosis. There was no significant change in Medicaid dental expenses.
70	Zwetchkenbaum, S. and J. Oh (2017). "More Rhode Island Adults Have Dental Coverage After the Medicaid Expansion: Did More Adults Receive Dental Services? Did More Dentists Provide Services?"	ACA Medicaid Expansion	RI Medicaid dental providers and patients	Retrospective analyses	RI Medicaid Management Information System	Number and percentage of receiving any dental service, dental claims by provider.	While the number of Medicaid dental services increased, the percentage of Medicaid beneficiaries receiving dental care decreased. Dental visits increased more in community health clinics compared to private clinics. Medicaid Expansion decreased the number of dentists submitting Medicaid claims, with the largest decrease occurring for dentists in private practice.
